# Supplementary material for: A glutamine sensor that directly activates TORC1
Source: Commun Biol. 2021 Sep 17;4:1093. doi: 10.1038/s42003-021-02625-w (PMC8448839; doi:10.1038/s42003-021-02625-w)
Supplement: Supplementary file 2 — Supplementary Information [file 42003_2021_2625_MOESM2_ESM.pdf]

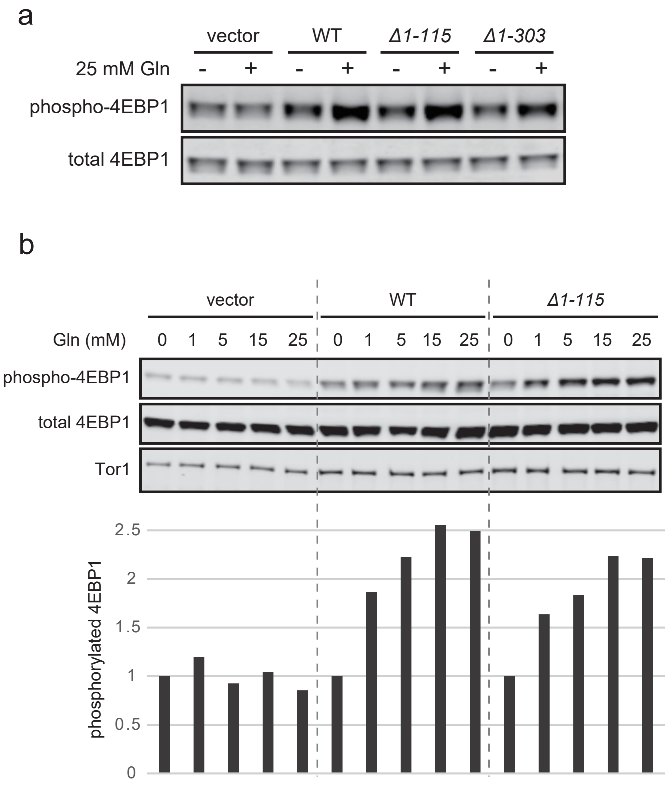

Supplementary Figure 1. Pib2(Δ1-115) responds to glutamine like wild-type Pib2.

a) In vitro kinase assays were performed (as for Fig. 1d) using permeabilized yeast cells prepared from *pib2Δ* cells (MH1059) carrying a vector (p416ADH) or plasmids encoding wild-type *PIB2* (pMH330), *pib2(Δ1-115)* (pMH334), or *pib2(Δ1-303)* (pMH410) with or without 25 mM L-glutamine. **b)** In vitro kinase assays were performed as in (**a**) with the indicated concentrations of L-glutamine. The bar graph shows the ratio of phosphorylated 4EBP1 to total 4EBP1, normalized to samples without glutamine.

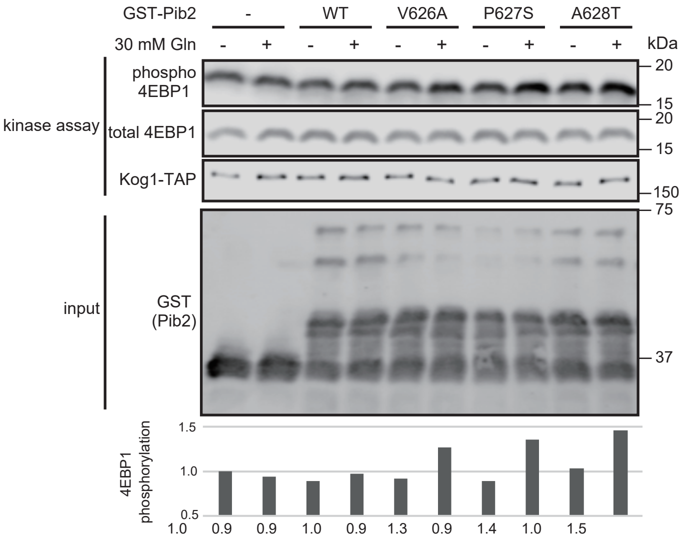

Supplementary Figure 2. Pib2 active mutants directly activate TORC1 in vitro.

An in vitro kinase assay was performed (as in Fig. 4) using purified TORC1 and bacterially -expressed Pib2 mutants. The bar graph shows the mean ratio of phosphorylated/total 4EBP1 normalized to sample with GST and without glutamine.

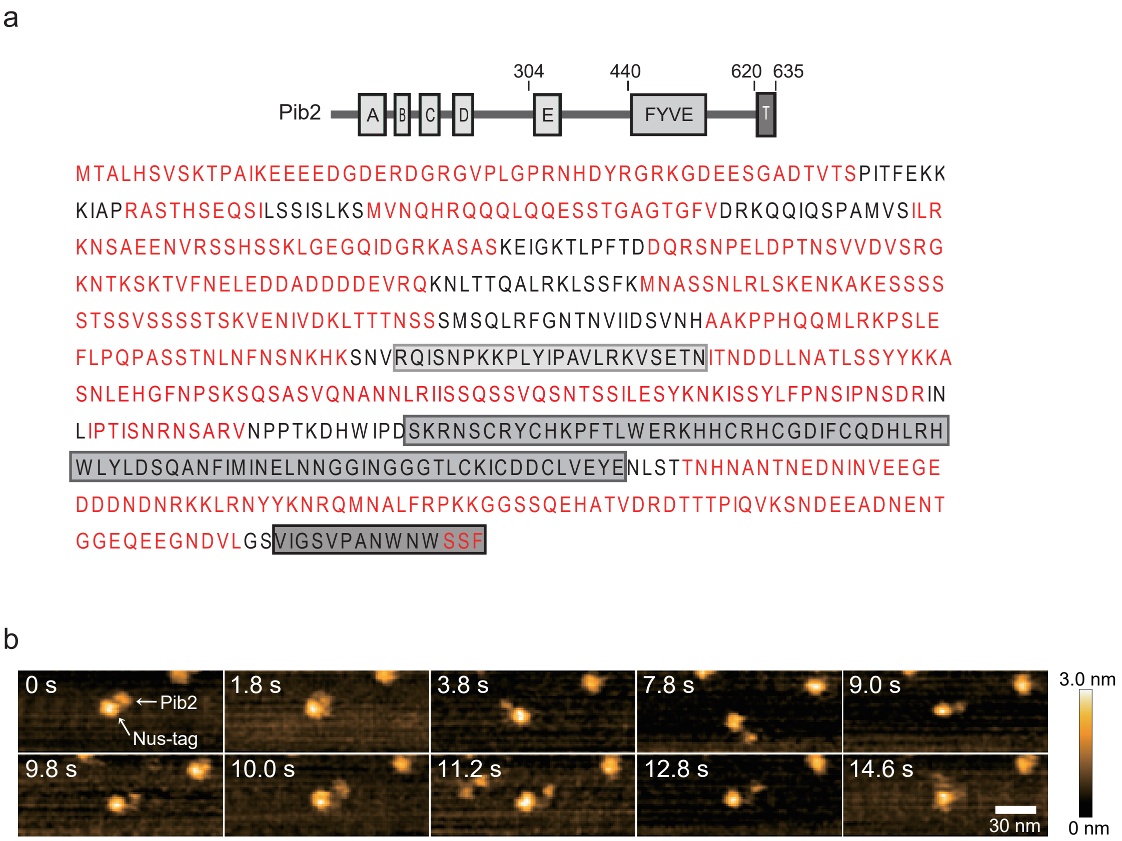

Supplementary Figure 3. Pib2 is an intrinsically disordered protein. a) Pib2 is predicted to have large intrinsically disordered regions by PrDOS (Ishida and Kinoshita, 2007). Predicted disordered regions are shown in red and the conserved E motif, FYVE domain, and tail motif are enclosed by squares. **b)** Successive HS-AFM images of NusA-tagged-Pib2(304-533, *Δ*356-384).

Uncropped western blot for Figure1b

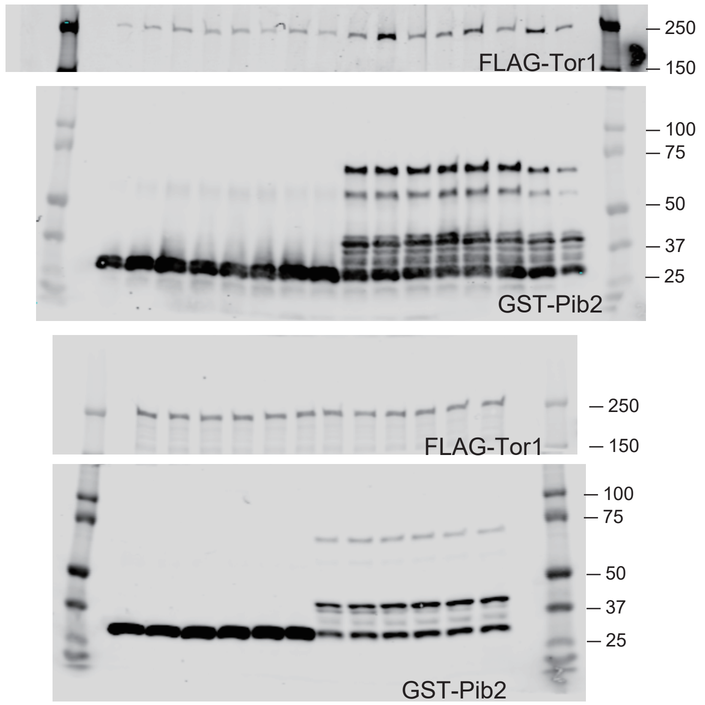

Uncropped western blot for Figure1c

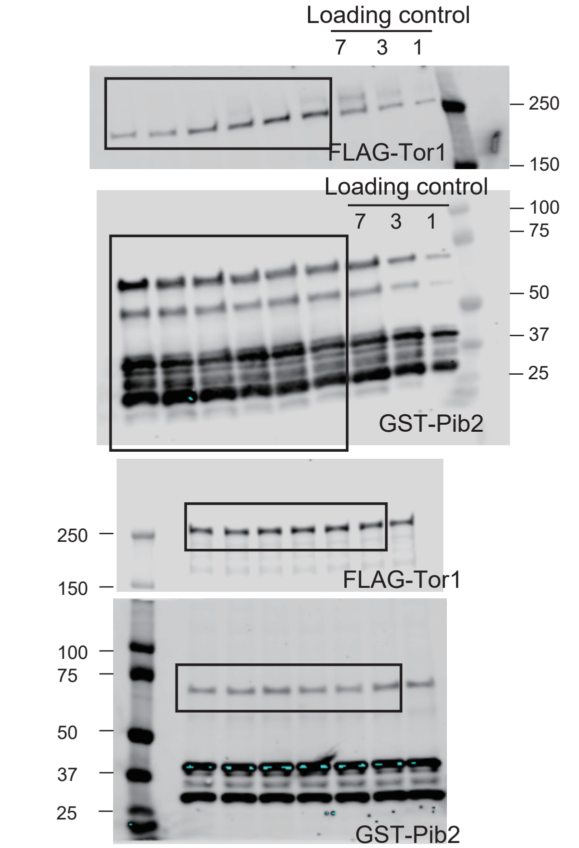

Uncropped western blot for Figure1d

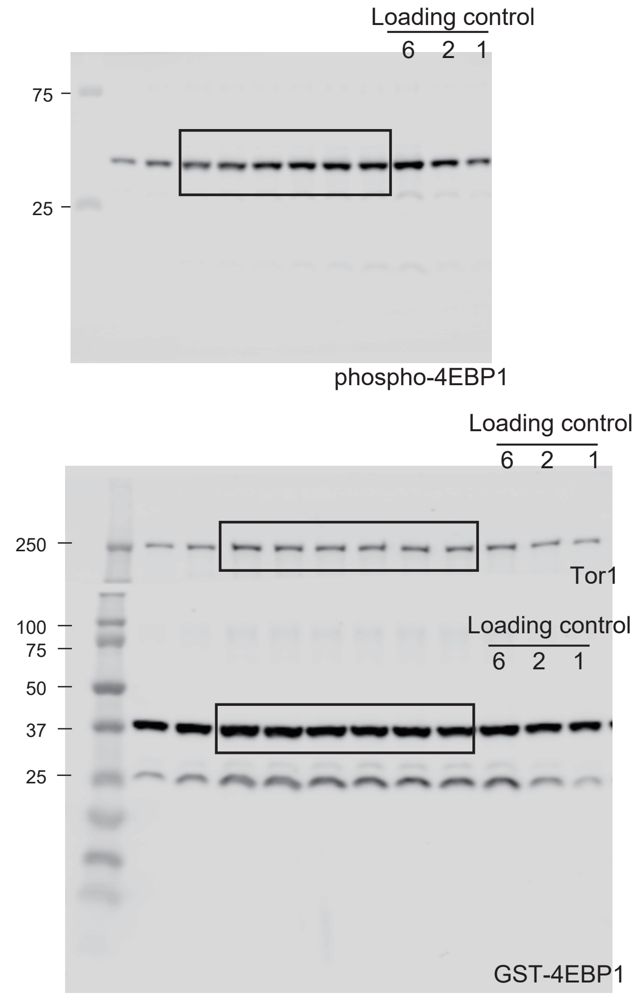

Uncropped western blot for Figure1f

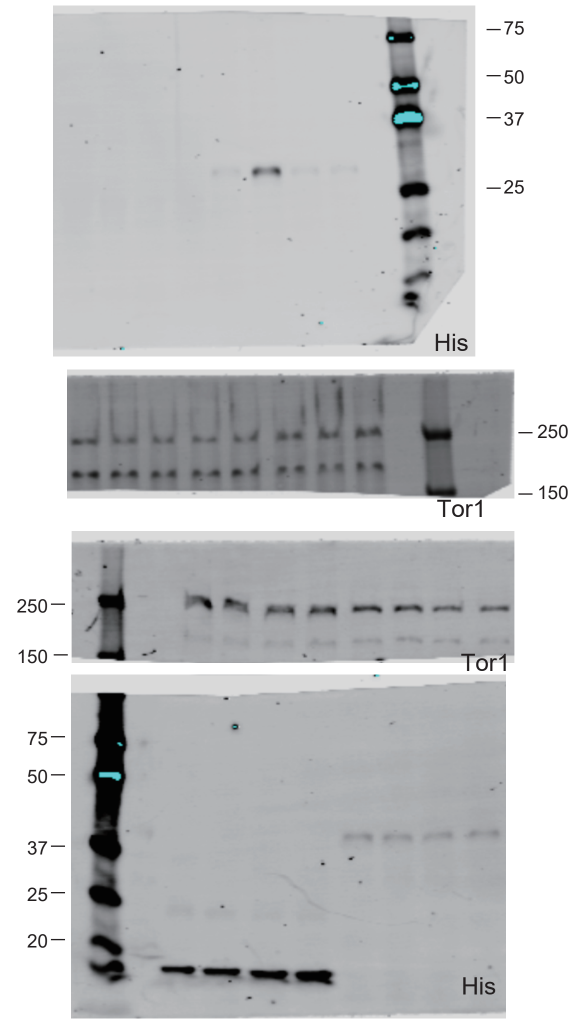

Supplementary Figure 4. Uncropped western blots.

Uncropped western blot for Figure1g

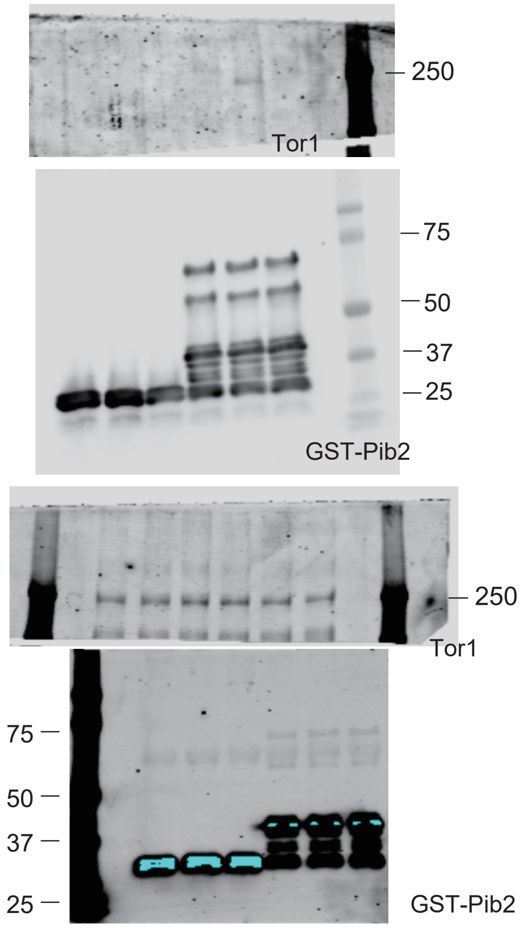

Uncropped western blot for Figure 2b

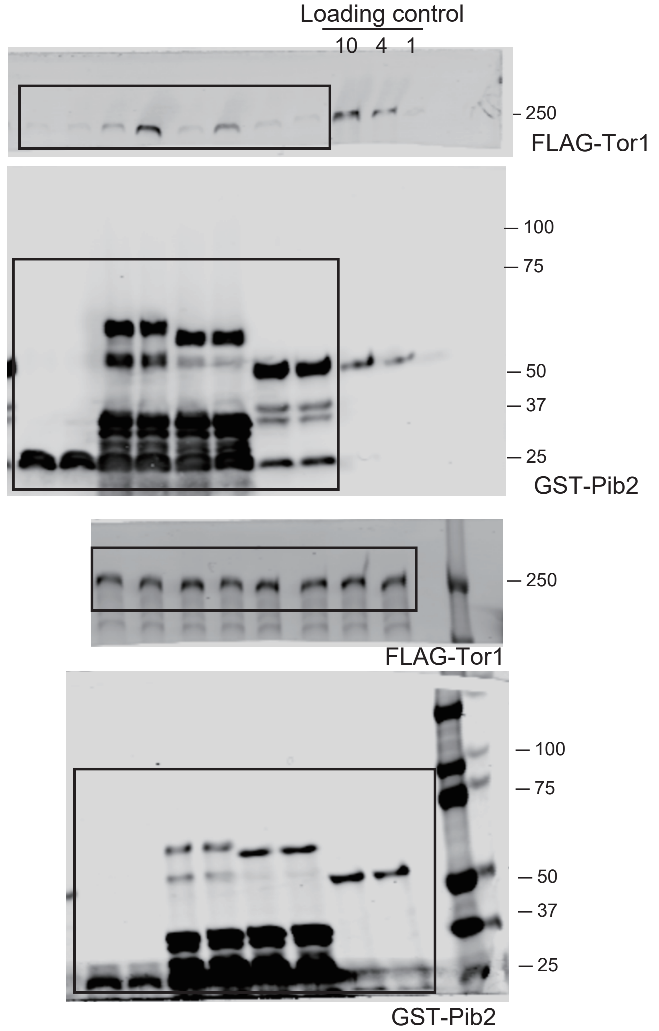

Uncropped western blot for Figure 2e

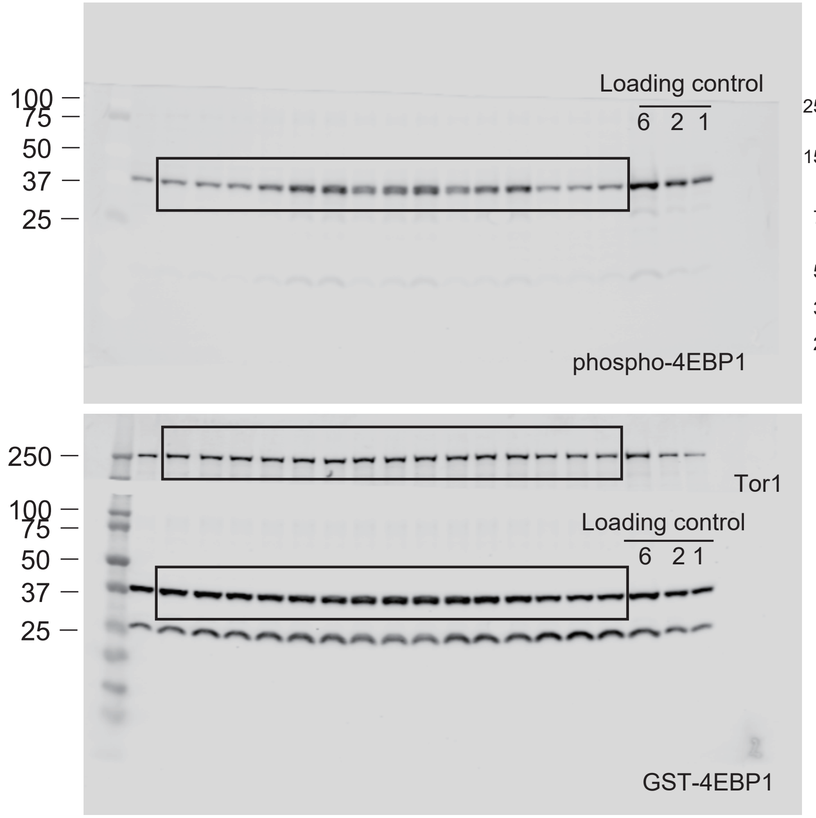

Uncropped western blot for Figure 2f

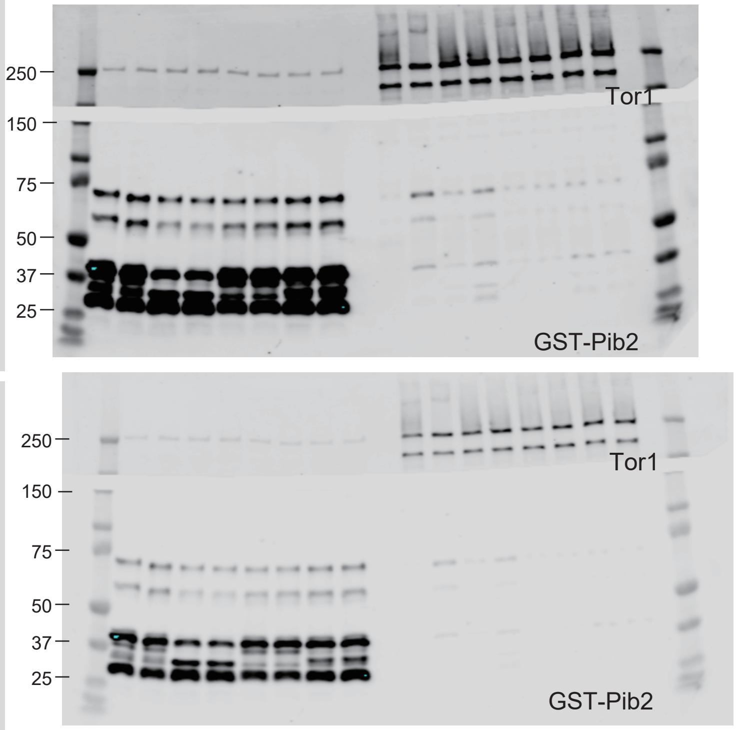

Supplementary Figure 5. Uncropped western blots.

Uncropped western blot for Figure3c

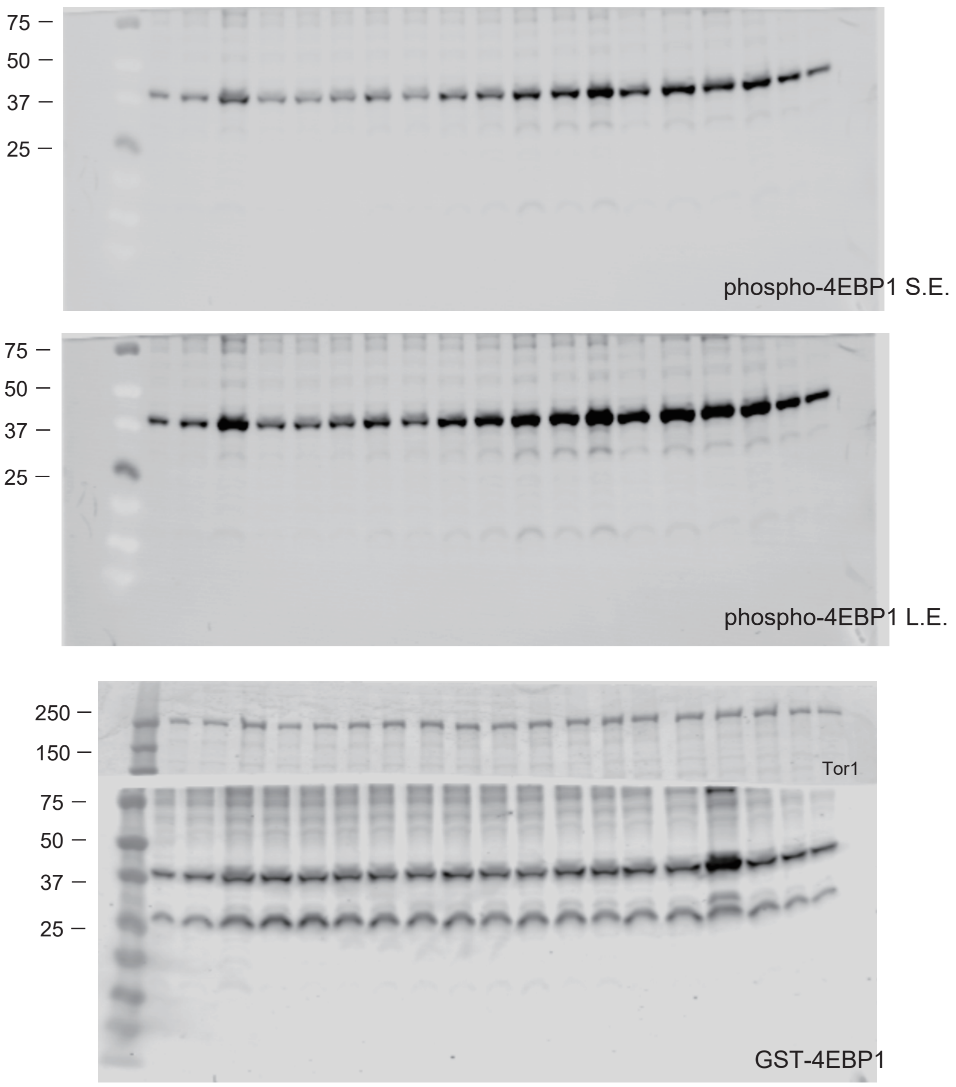

Uncropped western blot for Figure 3d

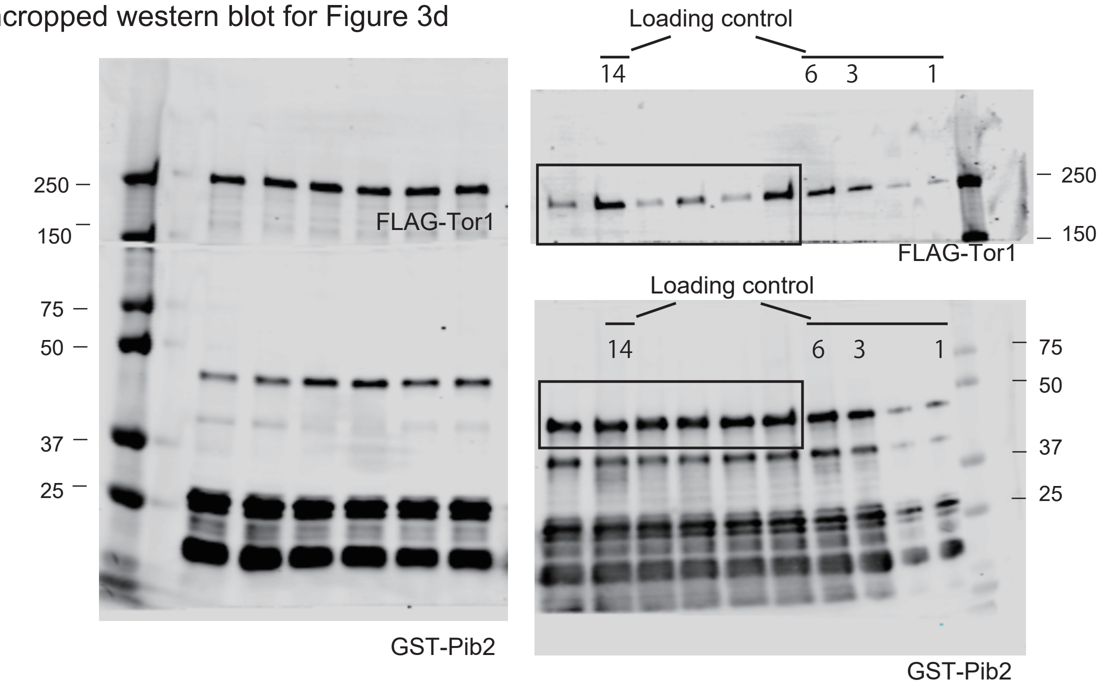

Supplementary Figure 6. Uncropped western blots.

Uncropped western blot for Figure 4

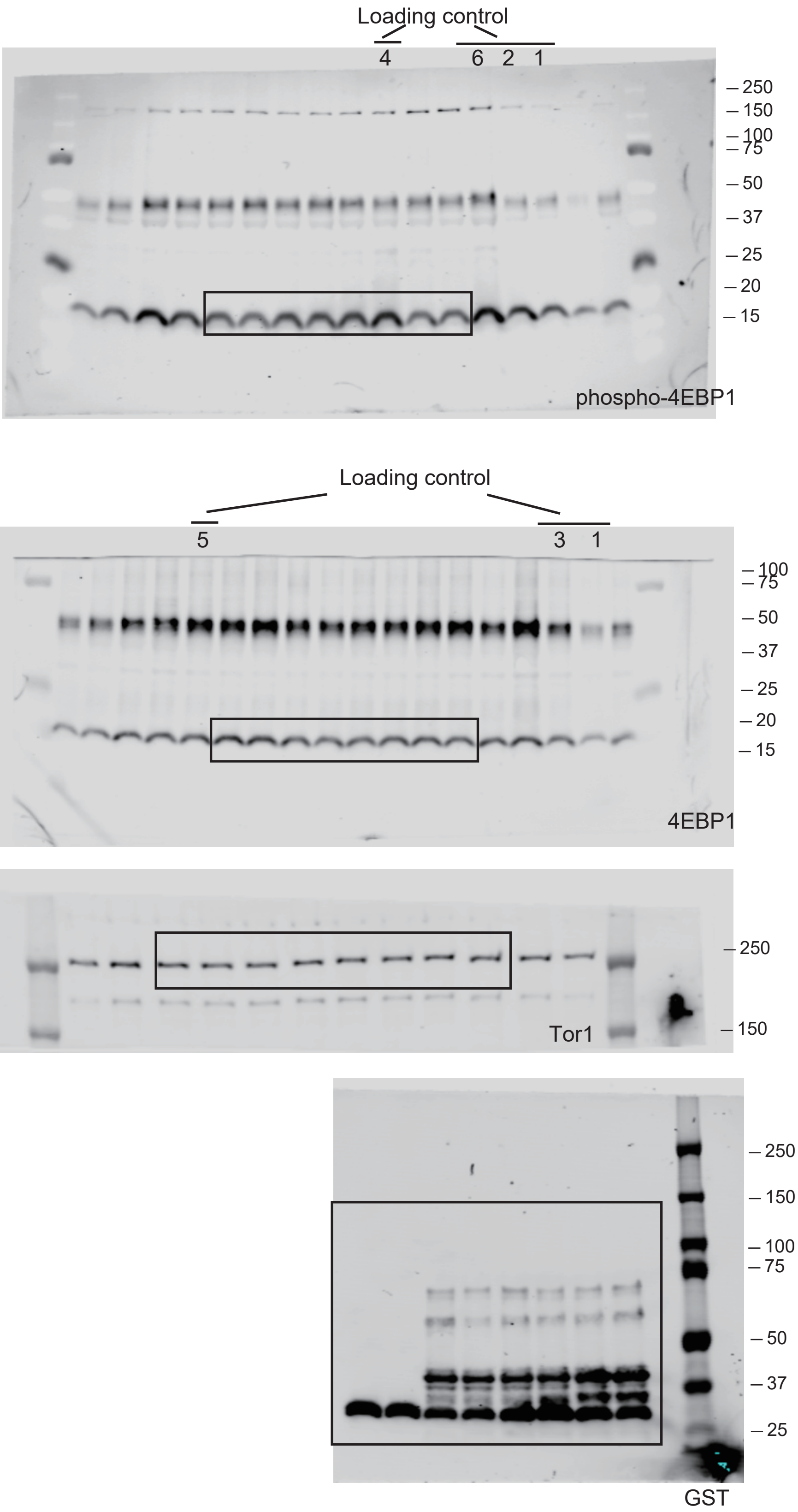

Uncropped western blot for Figure 5b

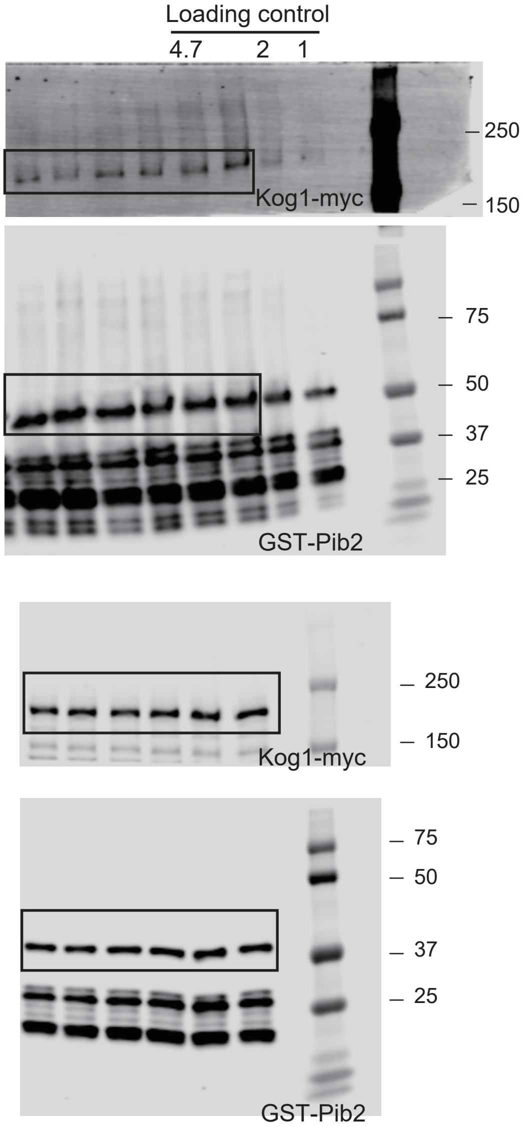

Supplementary Figure 7. Uncropped western blots.
