# Supplementary material for: A glutamine sensor that directly activates TORC1
Source: Commun Biol. 2021 Sep 17;4:1093. doi: 10.1038/s42003-021-02625-w (PMC8448839; doi:10.1038/s42003-021-02625-w)
Supplement: Supplementary file 3 — Reporting Summary [file 42003_2021_2625_MOESM3_ESM.pdf]

nature research

Corresponding author(s): Tatsuya Maeda

Last updated by author(s): Aug 11, 2021

Reporting Summary

Nature Research wishes to improve the reproducibility of the work that we publish. This form provides structure for consistency and transparency in reporting. For further information on Nature Research policies, see our Editorial Policies and the Editorial Policy Checklist.

Statistics

For all statistical analyses, confirm that the following items are present in the figure legend, table legend, main text, or Methods section.

n/a	Confirmed	
☐	☒	The exact sample size (*n*) for each experimental group/condition, given as a discrete number and unit of measurement
☐	☒	A statement on whether measurements were taken from distinct samples or whether the same sample was measured repeatedly
☐	☒	The statistical test(s) used AND whether they are one- or two-sided<br>*Only common tests should be described solely by name; describe more complex techniques in the Methods section.*
☒	☐	A description of all covariates tested
☒	☐	A description of any assumptions or corrections, such as tests of normality and adjustment for multiple comparisons
☐	☒	A full description of the statistical parameters including central tendency (e.g. means) or other basic estimates (e.g. regression coefficient) AND variation (e.g. standard deviation) or associated estimates of uncertainty (e.g. confidence intervals)
☒	☐	For null hypothesis testing, the test statistic (e.g. *F*, *t*, *r*) with confidence intervals, effect sizes, degrees of freedom and *P* value noted<br>*Give P values as exact values whenever suitable.*
☒	☐	For Bayesian analysis, information on the choice of priors and Markov chain Monte Carlo settings
☒	☐	For hierarchical and complex designs, identification of the appropriate level for tests and full reporting of outcomes
☒	☐	Estimates of effect sizes (e.g. Cohen's *d*, Pearson's *r*), indicating how they were calculated

Our web collection on statistics for biologists contains articles on many of the points above.

Software and code

Policy information about availability of computer code

Data collection	LI-COR Image studio ver.4.0
Data analysis	LI-COR Image studio ver.4.0, Photoshop, Excel 2016

For manuscripts utilizing custom algorithms or software that are central to the research but not yet described in published literature, software must be made available to editors and reviewers. We strongly encourage code deposition in a community repository (e.g. GitHub). See the Nature Research guidelines for submitting code & software for further information.

Data

Policy information about availability of data

All manuscripts must include a data availability statement. This statement should provide the following information, where applicable:
- Accession codes, unique identifiers, or web links for publicly available datasets
- A list of figures that have associated raw data
- A description of any restrictions on data availability

The datasets generated during and/or analysed during the current study are available in the figshare repository, https://figshare.com/s/fa8888dbd9d033075045.

Field-specific reporting

Please select the one below that is the best fit for your research. If you are not sure, read the appropriate sections before making your selection.

☒ Life sciences ☐ Behavioural & social sciences ☐ Ecological, evolutionary & environmental sciences

For a reference copy of the document with all sections, see nature.com/documents/nr-reporting-summary-flat.pdf

Life sciences study design

All studies must disclose on these points even when the disclosure is negative.

Sample size	The sample size was empirically determined to enable testing if differences between the samples are statistically significant.
Data exclusions	No data were excluded from the analysis, except for the failed western. The failure was judged by the loss of the linearity among the serially diluted positive controls on the same membrane.
Replication	The all attempts at replication were successful.
Randomization	No systematic randomization was used, although different sample allocation was generally used at replication experiments.
Blinding	Blinding was not appropriate in our study, because the ordering the samples was necessary for the time course experiments and the serial combinational experiments. Furthermore, the measurements automatically performed by the imaging system were essentially objective.

Reporting for specific materials, systems and methods

We require information from authors about some types of materials, experimental systems and methods used in many studies. Here, indicate whether each material, system or method listed is relevant to your study. If you are not sure if a list item applies to your research, read the appropriate section before selecting a response.

Materials & experimental systems

n/a	Involved in the study	
☐	☒	Antibodies
☒	☐	Eukaryotic cell lines
☒	☐	Palaeontology and archaeology
☒	☐	Animals and other organisms
☒	☐	Human research participants
☒	☐	Clinical data
☒	☐	Dual use research of concern

Methods

n/a	Involved in the study	
☒	☐	ChIP-seq
☒	☐	Flow cytometry
☒	☐	MRI-based neuroimaging

Antibodies

Antibodies used	The anti-Flag M2 antibody was from Sigma-Aldrich (St. Louis, MO, USA). The anti-Tor1 antibody yN-15 was from Santa Cruz Biotechnology (Dallas, TX, USA). The anti-myc antibody (9E10) was from Dr. Hoshikawa. The anti-phospho-4EBP1 (T37/46) antibody #9459 and anti-4EBP1 antibody #9452 were from Cell Signaling Technology (Danvers, MA, USA). The anti-GST antibody 4C10 was from Abcam (Cambridge, UK). The anti-Penta His antibody was from QIAGEN (Hilden, Germany). IRdye800-conjugated anti-rabbit antibody was from Rockland (Limerick, PA, USA) and Alexa 680-conjugated anti-mouse and anti-goat antibody was from Thermo Fisher Scientific (Waltham, MA, USA).
Validation	Commercially available antibodies used in this study are validated by each manufacturers. We also validated all primary antibodies to specifically detect the target proteins by western blotting.
